# MicroRNAs Implicated in the Immunopathogenesis of Lupus Nephritis

**DOI:** 10.1155/2013/430239

**Published:** 2013-07-07

**Authors:** Cristen B. Chafin, Christopher M. Reilly

**Affiliations:** ^1^Department of Biomedical Sciences & Pathobiology, Virginia-Maryland Regional College of Veterinary Medicine, Virginia Polytechnic Institute and State University, Blacksburg, VA 24061, USA; ^2^Edward Via College of Osteopathic Medicine, Blacksburg, VA 24060, USA

## Abstract

Systemic lupus erythematosus (SLE) is an autoimmune disease characterized by the deposition of immune complexes due to widespread loss of immune tolerance to nuclear self-antigens. Deposition in the renal glomeruli results in the development of lupus nephritis (LN), the leading cause of morbidity and mortality in SLE. In addition to the well-recognized genetic susceptibility to SLE, disease pathogenesis is influenced by epigenetic regulators such as microRNAs (miRNAs). miRNAs are small, noncoding RNAs that bind to the 3′ untranslated region of target mRNAs resulting in posttranscriptional gene modulation. miRNAs play an important and dynamic role in the activation of innate immune cells and are critical in regulating the adaptive immune response. Immune stimulation and the resulting cytokine milieu alter miRNA expression while miRNAs themselves modify cellular responses to stimulation. Here we examine dysregulated miRNAs implicated in LN pathogenesis from human SLE patients and murine lupus models. The effects of LN-associated miRNAs in the kidney, peripheral blood mononuclear cells, macrophages, mesangial cells, dendritic cells, and splenocytes are discussed. As the role of miRNAs in immunopathogenesis becomes delineated, it is likely that specific miRNAs may serve as targets for therapeutic intervention in the treatment of LN and other pathologies.

## 1. Introduction

Systemic lupus erythematosus (SLE) is an autoimmune disease characterized by the loss of immune tolerance to nuclear self-antigens. The deposition of autoantibodies along the glomerular basement membrane results in immune complex-mediated glomerulonephritis [1]. Mesangial cells, the primary immunoregulatory cells in the renal glomerulus, become activated due to the deposition of ICs. This recruits macrophages, B cells, T cells, and dendritic cells (DCs) to the kidney. Activated macrophages, mesangial cells, and DCs induce the maturation and activation of infiltrating T cells, which further activate macrophages and increase the B cell response [2]. Lupus nephritis (LN) is the major cause of morbidity and mortality in patients with SLE, affecting up to 70% of SLE patients [3]. Histological features include increased numbers of mesangial cells, overproduction of extracellular matrix, and inflammatory cell infiltration, which can lead to the development of sclerosis and fibrosis [4]. Depending on the severity of disease, 10–30% of LN patients will progress to end-stage renal failure [5]. 

It has been shown that genetic predisposition coupled with known and unknown environmental factors contributes to the development of SLE [6, 7]. Epigenetic defects have also been shown to play an important role in LN pathogenesis [8–10]. Epigenetics, which includes microRNA (miRNA) regulation, refers to stable and heritable changes in gene expression that alter the phenotype but not the underlying DNA sequence itself. miRNAs are small, non-coding RNAs that endogenously regulate gene expression by partially binding to the 3′ untranslated region (UTR) of target mRNAs [11–14]. miRNAs contribute to diverse physiological and pathophysiological functions including cell developmental timing, cell cycle control, apoptosis, and carcinogenesis [15, 16]. Hematopoiesis is fine-tuned by miRNAs at virtually every step [17]. In the last decade, increasing evidence has shown that miRNAs are critical not only for the regulation of immune cell development but also for modifying innate and adaptive immune responses [18]. 

A computational analysis performed on 72 lupus susceptibility genes in humans and mice revealed that most lupus susceptibility genes contain numerous target sites for over 140 conserved miRNAs. Three miRNAs (miR-181, miR-186, and miR-590-3p) are predicted to target over 50% of all lupus susceptibility genes [19]. Several studies have suggested that miRNAs play a role in the pathogenesis of LN by altering proinflammatory mediator production, innate immune cell responses, lymphocyte function, and Toll-like receptor (TLR) and NF*κ*B signaling pathways [20–24]. For example, miRNAs can induce the expression of proinflammatory cytokines, dictating the magnitude and duration of the immune response [25, 26]. miRNA dysregulation can result from genetic variation, hormonal influences, environmental triggers, or even the proinflammatory environment itself [27]. Lipopolysaccharide (LPS) induces the expression of miRNAs and activates transcription factors that further regulate miRNA expression [28, 29]. LPS has been shown to induce the expression of several miRNAs including miR-9, miR-132, miR-146, miR-155, and miR-let-7a (let-7a) [30–33]. Dysregulated miRNA expression may represent an underlying trigger that induces multifactorial diseases such as SLE. 

As pathogenic miRNAs are identified in LN pathogenesis, treatment strategies aimed at altering miRNA expression or signaling pathways may be employed to ameliorate disease pathogenesis in patients with SLE. Determining a patient's miRNA expression profile from the blood or urine may allow the use of targeted therapies to specifically modulate abnormal miRNA expression patterns in individuals suffering from lupus. It has been well documented that lupus patients respond to immunosuppressive agents with varying degrees of efficacy [34]. This has presented a major challenge in selecting the most effective treatment option. Determining how particular therapeutics alter pathogenic miRNAs may ultimately provide a viable screening tool for specific, targeted therapy in SLE. In this review, we summarize the current data on miRNAs in the major immune cells as related to LN pathogenesis and examine the future directions in miRNA-based therapy for SLE.

## 2. miRNAs Broadly Implicated in Inflammatory Diseases

### 2.1. miR-21

miR-21 is induced upon inflammatory stimulation and is a key component of TLR, NF*κ*B, and signal transducer and activator of transcription (STAT) signaling pathways [35–37]. The 3′ UTR of *programmed cell death 4* (*PDCD4)* is a direct target of miR-21. PDCD4 is a proinflammatory protein that promotes NF*κ*B activation and suppresses production of the anti-inflammatory cytokine IL-10. Overexpression of miR-21 in LPS-stimulated murine macrophages blocked NF*κ*B activity, decreased PDCD4 production, and promoted the production of anti-inflammatory IL-10. PDCD4-deficient mice are protected from LPS-induced death, presumably by an IL-10-mediated reduction in NF*κ*B activation [38]. 

miR-21 has been implicated in the immunopathogenesis of numerous inflammatory diseases [39]. Using an *in vitro* model of diabetes, Kato et al. showed that miR-21 overexpression in glucose-stimulated mesangial cells prevented cell proliferation by downregulating *tumor suppressor phosphatase and tensin homolog *(*PTEN*), whose 3′ UTR contains a binding site for miR-21 [40, 41]. miR-21 expression is induced by STAT3, a transcription factor activated by IL-6. miR-21 inhibition of PTEN leads to increased NF*κ*B activation that is required to maintain the transformed state. miR-21, PTEN, NF*κ*B, IL-6, and STAT3 are dynamic players in the positive feedback loop linking inflammation to cancer [35].

### 2.2. miR-146a

miR-146a may contribute to lineage determination in T cells as it is one of the only miRNAs that is differentially expressed in highly purified subsets of murine Th1 and Th2 cells [42]. Lu et al. demonstrated that miR-146a knockout mice develop a fatal immune-mediated disease similar to Foxp3 knockout mice that are devoid of functional Treg cells [43]. Although miR-146a knockout mice have increased Treg cells, their suppressive function is impaired. Treg cells without miR-146a (or Foxp3) acquire the ability to produce proinflammatory cytokines such as interferon-*γ* (IFN-*γ*). miR-146a deficiency in Treg cells caused an increase in STAT1 production, a key transcription factor required for Th1 effector cell differentiation. Because miR-146a regulates Treg cell suppressor function, the authors suggest that miR-146a maintains an optimal threshold of cytokine receptor-dependent activation of transcription factors that are necessary to suppress Th1 responses [43].

Since miR-146a has been reported to be an important negative regulator of acute responses during the activation of innate immunity, it has been suggested to play a regulatory role in the pathogenesis of SLE. miR-146a is induced by TLR activation (via LPS stimulation) and by proinflammatory mediators including tumor necrosis factor-*α* (TNF-*α*) and IFN-*α* [30]. miR-146a negatively regulates type I IFN production and myeloid differentiation factor 88 (MyD88) pathway activation induced by TLR stimulation [44]. Upon LPS-stimulated induction, miR-146a directly decreases TRAF6 and IRAK1 production, two signal transducers in the NF*κ*B activation pathway whose 3′ UTRs contain multiple miR-146a target sequences [30, 45]. Therefore, miR-146a reduces or terminates the inflammatory response through a negative-feedback loop by downregulating *TRAF6* and *IRAK1*. 

Due to its importance in the control of inflammation, several studies have sought to determine if miR-146a-based therapy can improve disease outcome in lupus-prone animal models or human patients. It was recently shown that treatment with the anti-inflammatory drug calcitriol alters the expression of miR-146a in SLE patients. Sera miR-146a expression, which is downregulated in patients with SLE, was significantly increased in patients after treatment with calcitriol for 6 months [46, 47]. These findings suggest that the immunomodulatory effects exerted by calcitriol in patients with SLE may be due, in part, to alterations in miR-146a expression. In addition, sera levels of miR-146a may be used to monitor treatment response.

### 2.3. miR-155

Like miR-146a, miR-155 is vital to proper immune system functioning; it is highly expressed in Treg cells and is induced upon activation of T effector cells and myeloid cells [48, 49]. miR-155 is induced in macrophages in response to both bacterial and viral antigens, functions in the hematopoietic compartment to promote the development of inflammatory T cells, and is required for DC production of Th17-promoting cytokines [28, 50]. By developing miR-155-deficient mice, Rodriguez et al. found that miR-155 is required for the proper functioning of DCs, B cells, and T cells [51]. The DCs of miR-155-deficient mice were unable to effectively activate T cells, indicating a defect in antigen presentation or costimulatory function. As they aged, the lungs of miR-155-deficient mice showed increased airway remodeling due to the increased numbers of lymphocytes in bronchoalveolar lavage fluids. The authors noted that these changes are similar to the lung fibrosis that often complicates systemic autoimmune processes with lung involvement [51]. Zhou et al. examined the regulatory role of miR-155 in the regulation of plasmacytoid dendritic cell (pDC) activation and type I IFN production [52]. They found that miR-155 is upregulated upon TLR stimulation, providing another example of the link between stimulation, miRNA expression, and cellular activation. These studies show that miR-155 is broadly implicated in LN pathogenesis and dysregulated miR-155 expression may play various roles in pathophysiology by altering immune cell function [52].

## 3. LN-associated miRNAs in Tissues

### 3.1. Renal Tissue

miRNA expression profiles of renal tissue have gained much attention since Dai et al. provided a broad analysis of differentially expressed miRNAs in LN kidney biopsies [53]. They identified 36 upregulated and 30 downregulated miRNAs in LN renal tissue compared to healthy controls. Their previous studies had identified 16 differentially expressed miRNAs in the peripheral blood mononuclear cells (PBMCs) of SLE patients, none of which constituted any of the 66 miRNAs identified in SLE kidney biopsies. These studies suggest that miRNA expression patterns are cell and organ specific [53, 54]. 

Lupus rodent models have revealed miRNAs implicated in LN pathogenesis (Table 1). In the anti-Thy1.1 rodent model of glomerulonephritis, TGF-*β* and other cytokines and growth factors promote mesangial cell proliferation and activation, leading to mesangial proliferative glomerulonephritis [55]. Denby et al. identified 2 miRNAs (miR-21 and miR-214) that are induced upon transforming growth factor-*β* (TGF-*β*) stimulation *in vitro* and characterized them further using the anti-Thy1.1 rat model [56]. TGF-*β*-induced overexpression of miR-21 and miR-214 in tubular epithelial cells caused epithelial-mesenchymal transition- (EMT-) like changes characterized by decreased *E-cadherin* expression and increased *α*-smooth muscle actin (**α*-SMA*) and *collagen type I* expression. These changes are characteristic of proliferating cells and tissue remodeling [57]. Blocking TGF-*β* downstream signaling in rat epithelial cells decreased the expression of miR-21 and miR-214 and prevented TGF-*β*-induced EMT by increasing *E-cadherin* expression and decreasing **α*-SMA* and *collagen type I* expression. These results suggest that TGF-B-induced miR-21 and miR-214 expression may contribute to extracellular matrix production and mesangial proliferative glomerulonephritis [56].

miRNAs that contribute to inflammation in chronic kidney disease (CKD) were recently examined in the B6.MRLc1 model, a congenic strain carrying a region of chromosome 1 derived from MRL/MpJ mice that develop IC-mediated glomerulonephritis [58]. miR-146a expression was found to be significantly increased in B6.MRLc1 kidneys compared to healthy controls. B6.MRLc1 mice that expressed high levels of miR-146a expression showed severe glomerular and interstitial lesions including cell infiltration, tubular atrophy, and interstitial fibrosis. The lesions had increased macrophage and T cell infiltration as well as increased expression of cell-specific mRNAs associated with the development of renal lesions (*CD68* and *S100a4* for macrophages and fibroblasts, resp.) [59]. miR-146a expression was also positively correlated with *IL-1*β**, *IL-10*, and *CXCL* expression. Because miR-146a is increased in the kidneys of B6.MRLc1 mice and continues to increase as they age, this model may be predisposed to increased miR-146a expression that initiates and perpetuates renal inflammation [59].

Research by Lu et al. confirmed that miR-146a is upregulated in glomerular tissue from LN patients and found that miR-146a is not overexpressed in LN tubulointerstitial tissue [60]. miR-638 expression, on the other hand, was underexpressed in glomerular tissue but higher in tubulointerstitial tissue. Glomerular expression of miR-146a positively correlated with both estimated glomerular filtration rate (GFR) and histological activity index, determined from the sum of semiquantitative scores of inflammation parameters [61]. Increased tubulointerstitial expression of miR-638 was positively correlated with proteinuria and SLE Disease Activity Index (SLEDAI) score. While the correlation between changes in miRNA expression and clinical disease severity suggests that these miRNAs may play a role in the pathogenesis of LN, it is currently unknown whether changes in miR-638 expression are pathogenic or an epiphenomenon [60]. 

### 3.2. PBMCs

While miR-146a was not initially reported to be decreased in SLE PBMCs, other miRNA expression screenings have revealed that miR-146a is significantly decreased in SLE patients and is inversely correlated with SLEDAI and IFN-*α*/*β* scores in SLE patients [46, 54, 62]. Furthermore, *in vitro* studies by Tang et al. revealed that overexpression of miR-146a reduced type I IFN induction in PBMCs [62]. They found that miR-146a negatively regulates both type I IFN production and TLR-stimulated downstream pathway activation by targeting the 3′ UTR of *interferon regulatory factor-5* (*IRF5*) and *STAT1*, key components in the type I IFN signaling cascade. The authors concluded that miR-146a deficiency is one of the causal factors in the abnormal activation of the type I IFN pathway in SLE [62].

A follow-up study identified a functional variant in the promoter of miR-146a that is associated with SLE disease risk; the promoter mutation resulted in decreased binding to the transcription factor ETS-1. Intriguingly, genomewide association studies have identified an association between SLE risk and a functional variant of *ETS1*. The researchers observed additive effects of the risk alleles of miR-146a and *ETS1*, which suggests that individuals with 2 or more of these alleles are at a greater risk of developing SLE than those carrying only one allele [63]. 

Stagakis et al. identified 27 differentially expressed miRNAs in the PBMCs of SLE patients, 2 of which corresponded with the miRNAs identified by Dai et al. and 19 of which correlated with disease activity [23, 54]. Of these disease-correlated miRNAs, eight were differentially expressed in T cells and 4 in B cells. Upregulation of miR-21 strongly correlated with disease activity and activated T cells; inhibition of miR-21 reversed the activated T cell phenotype by increasing *PDCD4* expression [23]. Another recent study found 7 abnormally expressed miRNAs (miR-145, miR-224, miR-150, miR-483-5p, miR-513-5p, miR-516a-5p, and miR-629) in SLE T cells compared to healthy controls. In a larger follow-up study, underexpression of miR-145 was confirmed and increased levels of *STAT1*, a target of miR-145, were observed in SLE T cells compared to healthy controls. Overexpression of miR-224 and decreased expression levels of its target, *apoptosis inhibitory protein 5* (*API5*), were also confirmed. T cells transfected with miR-224 *in vitro* were more susceptible to activation-induced apoptosis, indicating that SLE T cells overexpressing miR-224 may have an intrinsic defect that causes accelerated cell activation-induced apoptosis [64].

An additional study examining PBMC miRNAs found that decreased miR-125a expression in SLE patients contributed to increased KLF13 production by T cells. miR-125a has binding sites in the 3′ UTR of *KLF13*, which belongs to the family of transcription factors that regulates the expression of the inflammatory chemokine *RANTES* (*CCL5*) in T cells. Increased *RANTES* expression is associated with persistent or recurrent organ inflammation due to its recruitment of T cells to inflammatory tissues. Increasing miR-125a levels in T cells from SLE patients *ex vivo* alleviated elevated *RANTES* expression. This study confirmed that underexpression of miR-125a contributes to the elevated expression of *RANTES* in SLE, increasing T cell recruitment to inflammatory tissues [65]. 

## 4. LN-associated miRNAs in Innate Immune Cells

The innate immune response provides the initial defense against infection by external pathogens and is predominantly mediated by macrophages, DCs, and neutrophils. The presence of pathogens is commonly detected by macrophage and DC TLRs that bind conserved microbial products, triggering downstream signaling pathways to initiate inflammatory responses [66]. Through TLR activation, ICs from lupus patients induce pDCs to secrete type I IFN [67]. Activated DCs induce maturation and activation of infiltrating T cells, which further activates macrophages and increases the B cell response. The innate immune response, in particular DCs, promotes the activation of the adaptive immune system [68]. Since miRNAs are critical for modifying innate and adaptive immune responses, dysregulated miRNA expression may represent an underlying cause to LN pathogenesis (Table 1).

### 4.1. Macrophages/Mesangial Cells

miRNA expression is directly and indirectly altered after TLR activation and regulates macrophage signaling pathways that lead to the secretion of proinflammatory cytokines [28, 31, 75]. Let-7a and miR-147 are directly induced upon LPS stimulation due to NF*κ*B binding sites in their promoter regions, which induces the expression of proinflammatory cytokines including TNF-*α* and IL-6 [29, 75]. TNF-*α*, a critical cytokine involved in the response to LPS stimulation, increases miR-155 expression via JNK pathway activation, further increasing TNF-*α* production [28, 76, 77]. Inhibition of JNK blocks the induction of miR-155, demonstrating that upregulated miR-155 expression in LPS-stimulated macrophages is indirectly due to JNK pathway activation [31, 75]. These well-defined positive feedback loops demonstrate that stimulation-dependent miRNA expression induces cytokine production that further activates cells, which continues to alter miRNA expression. 

Mesangial cells, the primary immunoregulatory cells resident to the renal glomerulus, possess phagocytic and contractile properties. Regulatory mechanisms of mesangial cells include a complex array of factors that control cell proliferation, survival, apoptosis, and GFR. Mesangial cells from LN patients and lupus-prone mice have a heightened response to inflammatory stimulation [78, 79]. Mesangial cells from NZB/W mice have been shown to produce significantly more chemokines in response to LPS stimulation than controls [80]. Kato et al. demonstrated the involvement of miRNAs in mesangial cell activation [41]. They determined that TGF-*β* activates Akt in glomerular mesangial cells by inducing miR-215a and miR-217, revealing a role for miRNAs in kidney disorders. We recently found that let-7a expression was significantly increased in the mesangial cells of prediseased and actively diseased New Zealand Black/White (NZB/W) mice compared to age-matched New Zealand White (NZW) mice. Using *in vitro* techniques, we demonstrated that let-7a has a key role in regulating IL-6. Overexpression of let-7a increased IL-6 production in stimulated mesangial cells compared to nontransfected controls. Increased let-7a expression in the prediseased and diseased state may contribute to the increase in IL-6 production in young and old NZB/W mice. These data suggest that increased let-7a expression may predispose lupus mice to increased inflammatory mediator production with immune stimulation [32].

### 4.2. Dendritic Cells

Another significant immune cell that contributes to immunity in complex ways is the dendritic cell. DCs are widely considered to be critical for activating T cell responses, promoting B cell antibody production, and secreting cytokines in response to infections [81]. In these ways they may direct autoimmunity and tolerance by serving as the primary antigen presenting cells (APCs) to initiate T cell autoimmunity, promoting B cell autoantibody production, and secreting proinflammatory cytokines. Altered function of DCs is known to play a major role in the development of autoimmunity [82]. A recent study examining the functional characteristics of DCs in lupus patients found a significant increase in the percentage of cytokine-producing DCs in addition to an increase in the amount of cytokine per cell in SLE patients compared with healthy subjects [83]. pDCs are a specialized subset of DCs that are very active in IFN-*α* production, which promotes B cell differentiation into antibody-producing plasma cells (among many other functions). LN patients have been shown to have increased numbers of pDCs in the kidney as well as sustained IFN-*α* production [84].

The importance of TLR-induced miRNA expression in the regulation of pDC activation and type I IFN production has been examined. miR-155 and miR-155* (the complementary passenger strand in the miRNA duplex) were found to be the most strongly induced miRNAs in pDCs and were also differentially induced over time. The investigators found that miR-155* is induced before miR-155 and has biological activity. miR-155* induction after TLR stimulation increases IFN-*α* production by targeting *IRAKM*, which negatively regulates the TLR pathways by preventing the dissociation of IRAK1 and IRAK4 from MyD88 and the formation of IRAK1/TRAF6 complexes. The continual increase in miR-155 expression resulted in a reduction in IFN-*α* due to the targeting of *TAB2* by miR-155. TAB2 regulates type I IFN production in pDCs upon TLR stimulation. Taken together, these results suggest that there is cooperative involvement of both strands of the miRNA duplex in pDC activation [52].

## 5. LN-associated miRNAs in Adaptive Immune Cells

miRNAs were shown to be essential for altering the adaptive immune response in studies that conditionally depleted the enzyme Dicer from T or B cells. Dicer cleaves pre-miRNAs into double-stranded RNA products (duplexes) once they reach the cytoplasm [85]. T cell Dicer depletion indicated that miRNAs regulate diverse aspects of T cell biology, including basic cellular processes such as proliferation and survival as well as cell lineage decisions and cytokine production during T helper cell differentiation [86]. Dicer depletion in B cells resulted in the complete developmental block of B cells in the pro- to pre-B cell transition, affecting antibody diversity. These results indicate that miRNAs are critical for modifying adaptive immune responses and that irregular miRNA expression may represent an underlying cause to LN pathogenesis (Table 1) [87]. 

### 5.1. Splenocytes

Although many miRNAs are expressed in T cell subsets, one study found 7 miRNAs (miR-16, miR-21, miR-142-3p, miR-142-5p, miR-150, miR-15b, and let-7f) account for almost 60% of all T cell miRNAs. These miRNAs (except for miR-21) were downregulated in effector T cells compared to naïve cells. Memory T cell expression was similar to the expression seen in naïve T cells. miR-21 expression was higher in effector and memory T cells compared to naïve T cells, indicating that miRNAs are differentially expressed in hematopoietic lineages. These results suggest that miRNAs may contribute dynamically to cell differentiation and the maintenance of cell identity [88].

It has recently been demonstrated that murine lupus models share a common disease-associated miRNA expression pattern despite strain differences in lupus susceptibility loci and clinical manifestation. In the MRL/*lpr* model, miR-146a was associated with disease development due to increased expression in splenocytes from 3-4-month-old mice compared to 1-month-old mice. miR-155 was found to be associated with disease development in both the MRL/*lpr* and the NZB/W models [74]. An additional study investigated the relationship between IFN-accelerated disease, miRNAs, and B cell subsets in NZB/W mice due to the acceleration of disease by type I IFN in this model. Splenic and plasma miR-15a levels were elevated in diseased mice compared to prediseased mice. Increased autoantibody levels were significantly correlated with increased miR-15a expression. The immunosuppressive B cell subset (B-10) was reduced following IFN treatment, yet it had the highest miR-15a expression that increased with disease development. miR-15a expression in the pathogenic B cell subset (B-2) only increased upon disease onset. Although it is currently unknown whether changes in miR-15a expression are pathogenic or an epiphenomenon, these results suggest that miR-15a is implicated in the development of SLE in NZB/W mice by directing the balance of splenic B cell subsets [69].

Pathogenic miRNAs have also been examined in the lymphocytes of B6.Sle123 mice. These mice spontaneously develop autoimmune disease characterized by autoantibodies, splenomegaly, and IC-mediated glomerulonephritis. They also have elevated ratios of CD4^+^ to CD8^+^ T cells. The expression of miR-21, which is upregulated in SLE T cells and has been shown to regulate apoptosis and cell proliferation pathways in part by targeting *PDCD4*, was found to be upregulated in B6.Sle123 splenocytes [23, 70]. Short-term inhibition of miR-21 *in vivo* resulted in an approximate 20% decrease in *PDCD4* expression in naïve CD4^+^ T cells compared to T cells from control mice. Long-term inhibition of miR-21 *in vivo* significantly reduced splenomegaly in B6.Sle123 mice compared to the controls. In addition, the number of Fas receptor-expressing splenic B cells and the CD4^+^ to CD8^+^ T cell ratio were reduced, which suggests that miR-21 inhibition skews the T cell ratio towards that of the non-autoimmune strain [70]. 

The overexpression of miR-148a has also been investigated in CD4^+^ T cells from patients with lupus as well as lupus-prone mice. Due to the importance of DNA methylation abnormalities in SLE pathogenesis, Pan et al. examined the roles of mir-21 and miR-148a in aberrant CD4^+^ T cell DNA hypomethylation [71]. miR-21 and miR-148a downregulated *DNA methyltransferase 1 (DNMT1)* expression* in vitro* and *in vivo*, decreasing DNMT1 production in T cells. Downregulation of *DNMT1* in CD4^+^ T cells contributes to lupus autoreactivity by inducing T cell DNA hypomethylation; this results in the overexpression of autoimmunity-associated genes including *lymphocyte function-associated antigen 1* (*LFA-1 *or* CD11a*) and *CD70* [89]. While a putative miR-148a binding site has been predicted in the 3′ UTR of *DNMT1*, there are no predicted binding sites for miR-21. The researchers discovered that miR-21 indirectly downregulated *DNMT1* expression by targeting its upstream regulator in the Ras-MAPK pathway, *RASGRP1*. Intriguingly, miR-148a directly downregulated *DNMT1* expression by targeting the protein coding region of its transcript. In addition, miR-21 and miR-148a induced the overexpression of methylation-sensitive, autoimmune-associated genes in CD4^+^ T cells including *CD70* and *LFA-1*. Furthermore, the investigators found that the effects were reversed when inhibitors of either miR-21 or miR-148a were transfected into CD4^+^ T cells isolated from SLE patients, implying that hypomethylation in CD4^+^ T cells can potentially be alleviated by inhibiting these miRNAs [71].

Another posttranscriptional modifier of *DNMT1*, miR-126, was found to be overexpressed in CD4^+^ T cells from SLE patients [72]. Its degree of overexpression negatively correlated with DNMT1 protein levels. In addition, the expression of miR-142-3p and miR-142-5p was reduced to less than half in SLE CD4^+^ T cells compared to CD4^+^ T cells from healthy controls. miR-126, miR-142-3p, and miR-142-5p are predicted to target genes associated with SLE, which implicates their aberrant expression in CD4^+^ T cells in LN pathogenesis. Overexpression of miR-126 in primary CD4^+^ T cells from SLE patients contributed to T cell autoreactivity by targeting *DNMT1*, while inhibition in SLE patients resulted in T and B cell inactivation. Overexpression of miR-126 in primary CD4^+^ T cells from healthy donors resulted in the demethylation and upregulation of autoimmunity-associated genes including *CD11a* and *CD70*, inducing T cell and B cell hyperactivity. These results demonstrate that overexpression of miR-126 can aberrantly induce splenocyte activity towards that of an autoimmune phenotype [72].

Decreased expression of miR-142-3p and miR-142-5p in SLE CD4^+^ T cells was confirmed in studies by Ding et al. [73]. *CD84* and *IL-10* are predicted targets of miR-142-3p, while *signaling lymphocytic activation molecule-associated protein* (*SAP*) is a potential target of miR-142-5p. When miR-142-3p was inhibited in CD4^+^ T cells from healthy donors, protein levels of CD84 and IL-10 increased. SAP protein production was decreased after inhibition of mir-142-5p. Inhibition in healthy donor CD4^+^ T cells caused T cell overactivation and B cell hyperstimulation. These results were reversed after transfection of the corresponding miRNA mimic. Overexpression in SLE CD4^+^ T cells decreased CD40L, inducible T cell costimulator (ICOS), IL-4, IL-10, and IL-21 protein levels, reduced T cell proliferation, and reduced IgG production compared to controls. These results indicate that reduced expression of miR-142-3p and miR-142-5p in CD4^+^ T cells of SLE patients contributes to T cell hyperactivity and B cell hyperstimulation [73].

## 6. Future Directions in LN Treatment 

miRNAs are being recognized as potential therapeutic targets in the treatment of LN and other diseases as increasing numbers are identified as specific disease-modifying agents and not merely disease correlates. Recent studies have shown that exogenously increasing let-7a, a well-known tumor suppressor that is downregulated in many types of cancer, is effective in treating tumorigenesis by decreasing cell migration, invasion, and proliferation *in vitro* and *in vivo* [90–94]. Intranasal let-7 administration reduced lung tumor formation in a murine model of lung cancer [95]. Tumorigenesis was suppressed in murine gastric cancer cells *in vivo* by overexpression of let-7a, which decreased cell proliferation by causing G_1_ arrest [96].

Lupus therapeutics have recently been recognized for their ability to alter miRNA expression levels [97, 98]. Once disease-associated miRNA expression is determined in patients with SLE, tailored therapies can be designed using immunosuppressant treatments that alter pathologic miRNAs. Examining miRNA expression profiles during the course of immunosuppressant therapy may more accurately assess treatment responsiveness. Since lupus susceptibility genes contain target sites for various miRNAs, future treatments may target multiple disease-associated miRNAs that synergistically contribute to LN pathogenesis. Additionally, pathogenic miRNA expression may be used to assess treatment feasibility. This will allow the use of targeted therapies to specifically modulate abnormal miRNA expression patterns in individuals suffering from lupus.

Circulating miRNAs have been used as diagnostic markers in the treatment and diagnosis of certain cancers [99, 100]. Since the discovery of dysregulated miRNA expression in the serum and urine of SLE patients, the interest in using miRNAs as noninvasive biomarkers has increased [54, 97]. One of the many advantages of using miRNAs as disease biomarkers is the availability of highly sensitive PCR detection methods and their low complexity compared with protein biomarkers [101]. In addition, pathogenic miRNAs may be able to detect early SLE disease onset before clinical, pathological findings arise. Assessing miRNA expression in different tissues may alter our organ-specific and systemic understanding of SLE. For example, detecting alterations in urinary miRNA expression may offer valuable information regarding changes in the glomerular microenvironment, while pathogenic alterations in PBMCs may reveal the global state of the SLE patient.

### 6.1. Therapeutic Modulation of miRNAs

Because of the vast and critical roles miRNAs perform in fundamental immune processes (and due to their dysregulated expression in many pathological conditions), they have become an increasingly attractive target for therapeutic modulation. While the endogenous delivery of miRNAs has had limited testing *in vivo*, the risk of altering unintentional targets remains high as a single miRNA can have multiple gene targets and these targets can have profound effects on a variety of miRNAs [102–104]. The solution to this potential problem may lie in targeting miRNAs broadly associated with SLE such as miR-146a [42, 60, 62, 74, 105]. Pan et al. therapeutically altered miR-146a levels using virus-like particles (VLPs) containing miR-146a, which were delivered via tail vein injection to lupus-prone BXSB mice [106]. After administration of the miR146a-containing VLPs, high levels of miR-146a were detected in PBMCs, lung, spleen, and kidney tissues from BXSB and control mice. miR-146a therapy significantly reduced autoantibody, IFN-*α*, IL-1*β*, IL-6, and total IgG production. Widespread restoration of miR-146a by VLPs was effective in ameliorating SLE progression in lupus-prone mice, providing a potential novel therapy for SLE treatment [106].

While the initial findings from studies that systemically increase miRNA levels are promising, a more effective treatment may utilize targeted delivery systems. A novel approach to manipulating mesangial miRNA expression alone could be employed by targeting the mesangial cell surface marker that is unique to the kidney glomeruli: Integrin *α*8 [107]. Scindia et al. revealed that this molecule can be used to effectively target immunoliposomes to mesangial cells by tail vein injection [108]. pDCs also possess a unique cellular marker: plasmacytoid dendritic cell antigen (PDCA) [109]. In this way, pDCs may be specifically targeted instead of all splenocytes or PBMCs, considering that a miRNA may not be differentially expressed in other cell types found in the spleen or peripheral blood. For example, while miR-146a is decreased in PBMCs, it is upregulated in murine Th1 cells compared to naïve T cells and Th2 cells [62, 110]. 

### 6.2. Tailored Therapy Based on the Patient's miRNA Profile

Although glucocorticoids are the first-line treatment for a wide range of autoimmune diseases, up to 30% of patients with SLE are steroid resistant, demonstrating persistent tissue inflammation despite treatment with high doses of steroids [111, 112]. Disease-associated miRNAs may become unique biomarkers that help determine the course of the patient's immunosuppressant therapy. The use of miRNAs as selection markers for disease treatment is underway in the treatment of ovarian cancer. Researchers found that let-7a expression was predictive of a patient's outcome after chemotherapy; let-7a expression differed substantially between the patients who did or did not respond to chemotherapy containing platinum and paclitaxel. The survival of patients with low let-7a expression was higher when they received platinum and paclitaxel in combination; patients with high let-7a expression did not have improved survival after adding paclitaxel to platinum-based therapy [113]. 

If disease-associated miRNAs are targeted, the treatment of SLE could be greatly improved. Steroid-resistant patients, amongst others, may benefit from tailored immunotherapy. Revealing miRNAs with therapeutic potential may provide insight in treating other inflammatory diseases as well. Polikepahad et al. showed that the inhibition of let-7 miRNAs in an experimental model of asthma *in vivo* profoundly inhibited the production of allergic cytokines and the disease phenotype, indicating that let-7a may be a potential therapeutic target in other diseases as well [114].

## 7. Conclusion

miRNAs are now recognized as key regulators of gene expression. A single miRNA, or even multiple miRNAs, may contribute to cell development and immunoregulation in diverse ways. Increasing evidence has shown that miRNAs are not only critical for the regulation of immune cell development but also for modifying innate and adaptive immune responses. Evidence suggests that miRNAs are involved in LN pathogenesis by altering innate immune cell responsiveness, lymphocyte function, proinflammatory mediator production, and TLR and NF*κ*B signaling pathways. 

Increasing evidence indicates that dysregulated miRNA expression in specific cell types contributes to LN immunopathogenesis. While it is becoming clear that miRNAs modulate components of inflammatory signaling cascades, it is not fully understood how miRNAs are regulated by different cell types in SLE. Overall, the possibility of altering miRNA expression in order to ameliorate disease remains promising. Studies that alter pathogenic miRNAs have shown that miRNA-based therapies have the potential for becoming therapeutic tools for the treatment of SLE and other diseases. As we learn more about the intricacies of miRNAs and epigenetics, targets for drug development will continue to emerge.

## Figures and Tables

**Table 1:** miRNAs implicated in LN pathogenesis.

Cell or tissue type	miR ID(s)	Origin	Strain	Expression	Results	Mechanism(s)	Reference
Renal	21 and 214	R	WKY (anti-Thy1.1)	↑	Expression is induced by TGF-*β* in tubular epithelial cells *in vitro* and in renal tissue *in vivo *	Unknown	[56]
					Overexpression in tubular epithelial cells *in vitro* decreased *E-cadherin* expression and increased *collagen type I* and **α*-SMA * expression	Unknown	[56]
	146a	M	B6.MRLc1	↑	Increased expression positively correlated with *IL-1*β**, *IL-10*, and *CXCL * expression, severe glomerular and interstitial lesions, and T cell and macrophage infiltration	Unknown	[59]
		H	—	↑	Glomerular expression positively correlated with estimated GFR and histological activity index	Unknown	[60]
	638	H	—	↑	Tubulointerstitial expression positively correlated with proteinuria and disease activity score	Unknown	[60]

PBMCs	21	H	—	↑	Strongly correlated with disease activity and activated T cells	Unknown	[23]
					Inhibition *in vitro* reversed the activated T cell phenotype by increasing *PDCD4* expression	The 3′ UTR of *PDCD4* is a target of miR-21	[23]
	125a	H	—	↓	Underexpression contributes to the elevated expression of *RANTES* (*CCL5*) in SLE, increasing T cell recruitment to inflammatory tissues	The 3′ UTR of the *RANTES* upstream regulator *KFL13* is a target of miR-125a, indirectly increasing *RANTES* expression	[65]
	145	H	—	↓	Decreased expression increased *STAT1* expression in SLE patients	The 3′ UTR of *STAT1* is a target of miR-145	[64]
	146a	H	—	↓	Inversely correlated with disease activity and IFN-*α*/*β* scores	Unknown	[62]
					Overexpression reduced the induction and downstream effects of type I IFN	The 3′ UTR of *IRF5* and *STAT1* are targets of miR-146a, reducing the induction of type I IFN	[62]
					Promoter variant associated with SLE disease risk	SLE-associated SNP (rs57095329) decreases miR-146a expression levels	[63]
					Positively correlated with GFR, CRP, and other renal function parameters; inversely correlated with proteinuria and SLEDAI	Unknown	[46]
	155	H	—	↓	Positively correlated with GFR, CRP, and other renal function parameters	Unknown	[46]
	224	H	—	↑	Increased expression accelerated T cell activation-induced cell death by suppressing *API5* expression in SLE patients	The 3′ UTR of *API5* is a target of miR-224	[64]

Mesangial cells	Let-7a	M	NZB/W	↑	Increased expression throughout the lifetime of NZB/W lupus mice; overexpression increased *IL-6 *expression and IL-6 production *in vitro *	The 3′ UTR of *IL-6* is a target of let-7a; the exact mechanism of let-7a is unknown	[32]

Dendritic cells	155	H	—	↑	Induced by TLR stimulation after miR-155*; overexpression of miR-155 in normal pDCs significantly decreased *IFN-*α**, *IFN-*β**, and *TNF-*α** expression	The 3′ UTR of the type I IFN regulator *TAB2* is a target of miR-155, indirectly decreasing IFN-*α* and IFN-*β*	[52]
	155*	H	—	↑	Induced by TLR stimulation before miR-155; overexpression of miR-155* in normal pDCs significantly increased *IFN-*α**, *IFN-*β**, and *TNF-*α** expression	The 3′ UTR of the negative IFN regulator *IRAKM* is a target of miR-155*, indirectly increasing IFN-*α* and IFN-*β*	[52]

Splenocytes	15a	M	NZB/W	↑	Increased expression after disease was accelerated by IFN administration; differentially expressed in B cell subsets	Unknown	[69]
	21	M	B6.Sle123	↑	Inhibition increased *PDCD4* expression in T cells and reversed splenomegaly, improving overall disease outcome	The 3′ UTR of *PDCD4* is a target of miR-21	[70]
		M and H	MRL-*lpr *	↑	Downregulated *DNMT1* expression in T cells	The 3′ UTR of the *DNMT1* upstream regulator *RASGRP1* is a target of miR-21, indirectly downregulating *DNMT1 *	[71]
	126	H	—	↑	Overexpression contributes to T cell autoreactivity by decreasing *DNMT1 *expression	The 3′ UTR of *DNMT1* is a target of miR-126	[72]
					Overexpression in healthy donors was sufficient for T cell autoreactivity and B cell hyperstimulation, while inhibition in SLE patients resulted in T and B cell inactivation	Unknown	[72]
	142-3p and 142-5p	H	—	↓	Underexpressed in SLE CD4^+^ T cells	Dysregulated DNA and histone methylation of the miR-142 promoter	[73]
					Underexpression in CD4^+^ T cells increased production of CD84, IL-10, and SAP	The 3′ UTR of *CD84* and *IL-10* are targets of miR-142-3p; the 3′ UTR of *SAP* is a target of miR-142-5p	[73]
					Inhibition in healthy donor CD4^+^ T cells caused T cell overactivation and B cell hyperstimulation, while overexpression in SLE CD4^+^ T cells had the opposite effect	Although CD84 and SAP stimulate T-B cell interactions, the exact mechanism of miR-142 is unknown	[73]
	146a	M	MRL/*lpr *	↑	Increased expression associated with disease development	Unknown	[74]
	148a	M and H	MRL/*lpr *	↑	Downregulated *DNMT1* expression in T cells	The protein coding region of the *DNMT1* transcript is a target of miR-148a	[71]
					Induced overexpression of autoimmune-associated, methylation-sensitive genes in CD4^+^ T cells including *CD70* and *LFA-1 *	Inhibition of *DNMT1* results in DNA hypomethylation and the overexpression of methylation-sensitive genes	[71]
	155	M	MRL/*lpr*, NZB/W	↑	Increased expression associated with disease development	Unknown	[74]

Abbreviations: H: human; M: mouse; R: rat.

↑: increased expression; ↓: decreased expression.

*α*-SMA: *α*-smooth muscle actin; API: apoptosis inhibitory protein; CRP: C-reactive protein; DNMT: DNA methyltransferase; GFR: glomerular filtration rate; IFN: interferon; IL: interleukin; IRAK: IL-1 receptor-associated kinase, IRF: interferon regulatory factor; NZB/W: New Zealand Black/White; PBMCs: peripheral blood mononuclear cells; pDC: plasmacytoid dendritic cell; SNP: single-nucleotide polymorphism; PDCD: programmed cell death; SLE: systemic lupus erythematosus; SLEDAI: SLE Disease Associated Index; STAT: signal transducer and activator of transcription; TGF-*β*: transforming growth factor-*β*; TTP: tristetraprolin.

## Abbreviations

H: Humans
M: Mice
R: Rats
↑: Increased expression
↓: Decreased expression
*α*-SMA: *α*-Smooth muscle actin
API: Apoptosis inhibitory protein
CRP: C-Reactive protein
DNMT: DNA methyltransferase
GFR: Glomerular filtration rate
IFN: Interferon
IL: Interleukin
IRAK: IL-1 receptor-associated kinase
IRF: Interferon regulatory factor
NZB/W: New Zealand Black/White
PBMCs: Peripheral blood mononuclear cells
pDC: Plasmacytoid dendritic cell
SNP: Single-nucleotide polymorphism
PDCD: Programmed cell death
SLE: Systemic lupus erythematosus
SLEDAI: SLE Disease Associated Index
STAT: Signal transducer and activator of transcription
TGF-*β*: Transforming growth factor-*β*
TTP: Tristetraprolin.
